# Catalytic and Physicochemical Evaluation of a TiO_2_/ZnO/Laccase Biocatalytic System: Application in the Decolorization of *Azo* and *Anthraquinone* Dyes

**DOI:** 10.3390/ma14206030

**Published:** 2021-10-13

**Authors:** Agnieszka Kołodziejczak-Radzimska, Joanna Zembrzuska, Katarzyna Siwińska-Ciesielczyk, Teofil Jesionowski

**Affiliations:** 1 Institute of Technology and Chemical Engineering, Faculty of Chemical Technology, Poznan University of Technology, Berdychowo, PL-60965 Poznan, Poland; katarzyna.siwinska-ciesielczyk@put.poznan.pl (K.S.-C.); teofil.jesionowski@put.poznan.pl (T.J.); 2 Institute of Chemistry and Technical Electrochemistry, Faculty of Chemical Technology, Poznan University of Technology, Berdychowo, PL-60965 Poznan, Poland; joanna.zembrzuska@put.poznan.pl

**Keywords:** titania, zinc oxide, immobilization, decolorization, laccase from *Trametes versicolor*, azo and anthraquinone dyes

## Abstract

A TiO_2_/ZnO oxide system was proposed as a support for the immobilization of laccase from *Trametes versicolor* (LTV). The obtained TiO_2_/ZnO/LTV biocatalytic system was then applied in the decolorization/degradation of C.I. Reactive Black 5 and C.I. Acid Green 25 dyes. The efficiency of immobilization was evaluated based on catalytic properties (Bradford method, oxidation reaction of 2,2-azino-bis(3-ethylbenzothiazoline-6-sulfonic acid)) and physicochemical (spectroscopic, porous, electrokinetic) analysis. The immobilization process was carried out with high performance (99.4%). Immobilized laccase retained about 40% of its activity in the whole analyzed temperature range and after 10 reaction cycles. Immobilization efficiency was also indirectly confirmed by the presence of characteristic functional groups (–C–H and –C–O), nitrogen and carbon on the TiO_2_/ZnO/LTV biocatalytic surface, identified by spectroscopic analyses. The increase in the surface area to 126 m^2^/g, change of isoelectric point (2.0) and zeta potential ranges (from +12.0 to −20.0 mV) after the immobilization process were also observed. The results show that the designed biocatalytic system enables the removal of acid dyes (C.I. Reactive Black 5 and C.I. Acid Green 25) with high efficiency (99% and 70%, respectively). Mass spectroscopy analysis indicated possible degradation products formed by the cleavage of N=N and C–N bonds.

## 1. Introduction

Catalytic materials have been intensively researched in recent years with regard to their high activity in hydrogen evolution, pollution degradation, CO_2_ remediation and environmental purification [1,2]. TiO_2_ and ZnO are well-known semiconductor catalysts offering stability and economic advantages [3,4]. TiO_2_ is an interesting candidate for environmental protection applications due to its non-toxicity, biological and chemical stability, strong oxidizing properties, low price and resistance to corrosion [5,6]. Titanium oxide is a common photocatalyst which is the subject of numerous studies relating to the removal of organic pollutants, due to its wide sourcing, rich content, stability in solution under light, and high photocatalytic ability under UV radiation [7]. However, the activity of TiO_2_ in the visible region is limited due to its wide band gap [8,9]. To improve its properties, hybrid systems based on TiO_2_ are developed. For example, TiO_2_ is combined with ZnO, which is non-toxic, cheap, easy to synthesize and has a broad spectrum of applications [10]. The preparation of such a hybrid system (TiO_2_/ZnO), with appropriately selected parameters of the synthesis process, can result in a product with strictly defined physicochemical parameters [11].

Industrial waste poses a significant threat to the natural environment and living organisms, and it is extremely important to reduce this impact by utilization or complete neutralization [12,13]. Toxic or carcinogenic organic compounds and the large quantities of this waste pose a significant problem [14]. Biological methods are among the methods used to remove inorganic and organic contaminants from wastewaters. They are an alternative method for the treatment of industrial wastewaters, in view of their availability, cost-effectiveness and eco-friendliness [15,16,17]. Biosorption, bioaccumulation and biodegradation, or a combination of these mechanisms, are the main processes used in such biological methods [18,19]. In the case of biodegradation, an enzyme is used to convert the pollutants to simpler compounds, which are usually less toxic [20,21]. Because of limitations on the use of native enzymes, an immobilization process is carried out. This increases thermal and pH stability and repeatability, making the enzymes better suited for environmental applications [22,23,24].

The selection of a suitable carrier is a significant factor in the process of enzyme immobilization. Numerous literature reports show that various materials, both organic, inorganic and hybrids, are used as carriers [25,26]. The inorganic materials, especially oxide compounds, offer good thermal and chemical stability, as well as excellent mechanical resistance. Moreover, they have a well-developed porous structure that provides a good environment for enzyme immobilization [27,28,29]. Both TiO_2_ and ZnO are examples of compounds that possess the above-mentioned multifunctional properties.

ZnO and TiO_2_ have been widely used as supports for enzyme immobilization [30,31,32,33]. For example, Movahedi et al. [34] developed an easy and effective method of increasing the stability of lactoperoxidase (LPO) immobilized on ZnO nanoparticles (ZnO/LPO). The effectiveness of immobilization was affirmed by Fourier transform infrared spectroscopy (FTIR) and field emission scanning electron microscopy (FE-SEM). The results exhibited that the stability of the immobilized lactoperoxidase was meaningfully improved, compared with the free enzyme. ZnO/LPO retained 18% of its initial activity after 30 days at 25 °C. In turn, Husain et al. [35] immobilized β-galactosidase from *Aspergillus oryzae* on zinc oxide (ZnO) through a simple adsorption mechanism. ZnO with β-galactosidase retained about 68% of its initial activity. Due to its easy production, better stability against various chemical compounds, and excellent reusability, β-galactosidase immobilized on ZnO may find applications in the construction of analytical devices based on enzymes for use in clinical, environmental and food technologies. A hybrid system containing ZnO and SiO_2_ has also been used for enzyme immobilization. Shang et al. [36] utilized the ZnO/SiO_2_ system to immobilize lipase by an adsorption process. The immobilized enzyme demonstrated preferable thermal and chemical stability and reusability than the free lipase. The immobilized lipase was used as a biocatalyst for the synthesis of phytosterol esters by esterification of phytosterol and oleic acid, and a 96% yield was obtained under optimal conditions. After twelve periods, the immobilized enzyme retained 89% of its initial activity. In another study, TiO_2_ modified with different micro-environments was used as a carrier for penicillin G acylase (PGA) immobilization. The results demonstrated that the functional groups and arm-length of the immobilization sites of the titania had a good influence on the enzymatic yield of immobilized PGA [37]. Liu et al. [38] used lipase immobilized onto Fe_3_O_4_/TiO_2_ nanoparticles by electrostatic interaction. The immobilized enzyme showed a wider range of thermal and chemical stability, as well as better storage stability and reusability. The kinetic properties of the immobilized lipase were investigated. These studies helped develop a method for discovering new anti-obesity drugs. A binary and ternary oxide system based on TiO_2_ was used by Zdarta et al. for laccase immobilization [39]. The proposed materials have achieved high immobilization efficiency (about 90%). Other publications show that biocatalytic systems based on TiO_2_ or ZnO and laccase can also be efficiently applied in the removal of organic dyes (Alizarin Red S, Reactive Black, Malachite Green, Remazol Brilliant Blue R, etc.) from aqueous systems [40,41,42,43,44].

Summarizing the above-mentioned research, it is concluded that TiO_2_ and ZnO have been successfully used as carriers in enzyme immobilization processes. Following this path, in this work it was decided to develop a TiO_2_/ZnO material as a laccase carrier, combining the properties of both TiO_2_ and ZnO. The TiO_2_/ZnO oxide system, prepared via a sol-gel method, was used for the first time as a support for the immobilization of laccase from *Trametes versicolor*, and the resulting biocatalytic system (TiO_2_/ZnO/LTV) was then applied in the decolorization/degradation of C.I. Reactive Black 5 and C.I. Acid Green 25. The efficiency of immobilization was determined based on catalytic properties (Bradford method, oxidation reaction of ABTS) and physicochemical (spectroscopic, porous and electrokinetic) evaluation. Additionally, the influence of time, pH and temperature on the efficiency of the decolorization/degradation of the acid dyes was determined.

## 2. Materials and Methods

### 2.1. Materials

All of the materials used in the research were purchased from Sigma-Aldrich^®^ (St. Louis, MO, USA). Zinc acetate hydrate (ZnAc), titanium (IV) isopropoxide (TTIP), propan-2-ol (IP) and sodium hydroxide (NaOH) were used to fabricate the TiO_2_/ZnO support. Laccase from *Trametes versicolor* (LTV), Bradford reagent, buffer solution, 2,2-azino-bis(3-ethylbenzothiazoline-6-sulfonic acid) (ABTS) were utilized in the immobilization process. C.I. Reactive Black 5 (RB5) and C.I. Acid Green 25 (AG25) were used in the dye decolorization (Table 1). Laccase is a blue copper oxidase of white rot fungus, *Trametes versicolor*, that reduces molecular oxygen to water. Most of the information about laccase from *Trametes versicolor* originates from a species belonging to Basidiomycota. In addition, the active center of this enzyme is the tricopper supramolecular site, which has a catalytic effect leading to the degradation of dyes.

### 2.2. Details of TiO_2_/ZnO Synthesis, Laccase Immobilization, Catalytic Studies and Physicochemical Analysis

**Figure 1:**
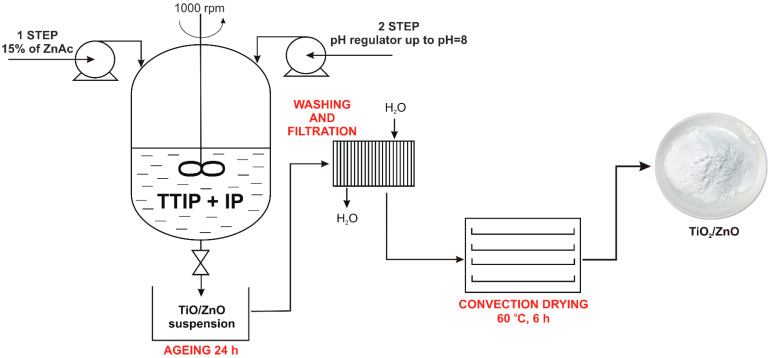
Schematic diagram of the preparation of TiO_2_/ZnO.

In the first step of the research, the TiO_2_/ZnO system was prepared according to the method proposed by Siwińska-Ciesielczyk et al. [45]. In this method, zinc acetate (ZnAc) and titanium isopropoxide (TTIP) were used as precursors of Zn and Ti oxides. The scheme of the TiO_2_/ZnO preparation process is shown in Figure 1. Appropriate amounts of propan-2-ol (IP, 25 mL) and titanium (IV) isopropoxide (TTIP, 10 mL) were immersed in the reactor. Next, zinc acetate (15% wt., 50 mL) was dosed into the reactor (5 mL·min^−1^), and the pH regulator (1 mol·L^−1^ NaOH) was added to achieve pH = 8. The reactor was equipped with a stirrer, which was used to homogenize the reaction system (1000 rpm). The next step of the synthesis was crystallization (ageing, 24 h). The resulting sediment (TiO_2_/ZnO) was washed by water and filtered, and then dried at 105 °C.

In the second stage, the adsorption immobilization of laccase onto TiO_2_/ZnO was performed. The TiO_2_/ZnO (0.5 g) was mixed with the laccase solution (25 mL, 5 mg/mL in 0.1 mol·L^−1^ acetate buffer at pH = 4). That process was performed in an incubator (IKA-Werke, Staufen, Germany) for 24 h at 20 °C. To define the immobilization effectiveness, Bradford analysis was performed [46]. On that basis, the amount of immobilized laccase (*P*, mg/g_support_) and the immobilization efficiency (*IY*, %) were determined. The quantity (*P*, mg/g) of laccase immobilized onto TiO_2_/ZnO and the immobilization yield (*IY*, %) were calculated using the Bradford method and following Equations (1) and (2):

(1) $$P=\frac{\left(C_{0}-C_{1}\right)\times V}{m}$$

(2) $$IY=\frac{C_{1}}{C_{0}}\times 100\%$$

where *C*_0_ and *C*_1_ signify the concentration of the enzyme (mg/mL) in the solution before and after immobilization, respectively, *V* is the volume of solution (mL), and *m* is the mass of TiO_2_/ZnO (g).

The obtained biocatalytic system TiO_2_/ZnO/LTV was used as a catalyst in the oxidation of 2,2-azinobis-3-ethylbenzthiazoline-6-sulphonate (ABTS). This reaction was led through adding the immobilized laccase (approximately 10 mg) into 0.1 mM ABTS (pH 4.0, 20 mL) at 40 °C. Next, the absorbance was measured at λ = 420 nm, using a Jasco V-750 spectrophotometer. Based on that reaction, the influence of the pH, temperature, storage and reuse on the enzymatic activity of TiO_2_/ZnO/LTV was also investigated. All measurements were done in triplicate. The results are showed as mean ± 3.0 SD.

Additionally, several physicochemical analyses were done to determine the efficiency of the synthesis, immobilization and degradation. These included Fourier transform infrared spectroscopy (FTIR; Vertex 70 spectrometer, Bruker, Billerica, MA, USA), X-ray photoelectron spectroscopy (XPS; Prevac, Rogow, Poland), and the determination of porous parameters (ASAP 2020 instrument, Micromeritics Instrument Co., Norcross, CA, USA) and electrokinetic stability (ζ; Zetasizer ZS equipped with an MPT-2 automatic titration system, Malvern Instruments Ltd., Malvern, UK). To characterize the groups that confirm the presence of the enzyme on the TiO_2_/ZnO surface, the FTIR analysis (Fourier transform infrared spectroscopy) was undertaken. The samples were prepared in the form of KBr tablets. The analysis was done over a wavenumber range of 4000–400 cm^−1^. The surface composition of the obtained materials, before and after enzyme immobilization, was also analyzed by means of X-ray photoelectron spectroscopy, using Al Kα monochromatized radiation (hν = 1486.6 eV) with a Prevac system equipped with a Scienta R2002 electron energy analyzer perating at constant transmission energy (Ep = 50 eV). The samples were loosely situated into a grooved molybdenum holder. The experimental errors were estimated to be ± 0.1 eV for the photoelectron peaks of carbon and nitrogen. The surface area (*A_BET_*), total pore volume (*V_p_*) and mean pore diameter (*S_p_*) were designated based on low-temperature N_2_ sorption, Brunauer–Emmett–Teller and Barrett–Joyner–Halenda methods. All of the samples were degassed (support at 120 °C and biocatalytic system at 70 °C) for 4 h prior to measurement. The evaluation of electrophoretic mobility and determination of the zeta potential were based on the technique of Laser Doppler Velocimetry phenomenon (LDV). The electrophoretic mobility was measured at a constant ionic strength of 0.001 M NaCl, and the zeta potential value was then calculated based on the Henry equation. First, 0.01 g of the appropriate material was placed in 10 mL of NaCl solution, and measurements were then carried out. Titration was performed with a 0.2 M solution of hydrochloric acid or sodium hydroxide.

### 2.3. Decolorization of Organic Dyes

The third stage of the research involved the decolorization of acid dyes (C.I. Reactive Black 5 and C.I. Acid Green 25). During this process, the influence of time (0.5, 1, 3, 6, 9, 12, 24 h), temperature (25–70 °C) and the pH of the environment (2–9) on the effectiveness of decolorization was identified. The dye decolorization process was performed using the immobilized LTV (TiO_2_/ZnO/LTV). An adequate amount of the TiO_2_/ZnO/LTV biocatalytic system (0.1 g) was added to the 10 mL of dye solution (50 mg/mL). The reaction was done in an incubator for 24 h at an ambient temperature. The mixture was then filtered under low pressure, and the solution underwent spectrophotometric analysis (Abs_AG_ = 605 nm, Abs_RB_ = 620 nm, V-750 spectrophotometer, Jasco, Oklahoma City, OK, USA). The decolorization efficiency was calculated based on the Equation (3):

(3) $$DE=\frac{C_{0}-C_{1}}{C_{0}}\times 100\%$$

where *DE* is the efficiency of decolorization of the organic dye, and *C*_0_ and *C*_1_ are the concentrations of dye before and after the decolorization process, respectively.

#### Mass Spectroscopy (MS) Analysis

MS analysis of the dyes and their reduced products confirmed the degradation of C.I. Reactive Black 5 and C.I. Acid Green 25 by enzymatic treatment. Sample solutions before analysis were diluted by methanol. Full scan ESI mass spectra were received on an API 4000 QTRAP mass spectrometer (AB Sciex, Foster City, CA, USA). Sample solutions were introduced into the ESI source using a syringe pump at a flow rate of 10 µL/min. The ESI was worked in positive (C.I. Reactive Black 5) and negative (C.I. Acid Green 25) ion mode. Dye solutions were detected using the following parameters: curtain gas at 10 psi, nebulizer gas at 45 psi, auxiliary gas at 45 psi, and temperature 400 °C. The ion spray voltage was 5500 or −4500 V and the declustering potential was 100 V. In order to achieve better readability of the peaks, 10 scans were averaged.

## 3. Results and Discussion

### 3.1. Catalytic Characterization of TiO_2_/ZnO/LTV

The immobilization process of laccase from *Trametes versicolor* on the TiO_2_/ZnO oxide system was carried out four times. Based on the four repetitions, it can be concluded that the process of laccase immobilization onto TiO_2_/ZnO is reproducible. The amount of immobilized laccase was 469.6 mg/g_support_. The results (presented in Table 2) show that the immobilization process was carried out with high efficiency (99.4%). Similar effects were received by Wang et al. [42], who used TiO_2_ sol-gel coated PAN/O-MMT (polyacrylonitrile/organically modified montmorillonite) as a laccase support. In that case, 342 mg of laccase was immobilized on 1 g of support.

**Figure 2:**
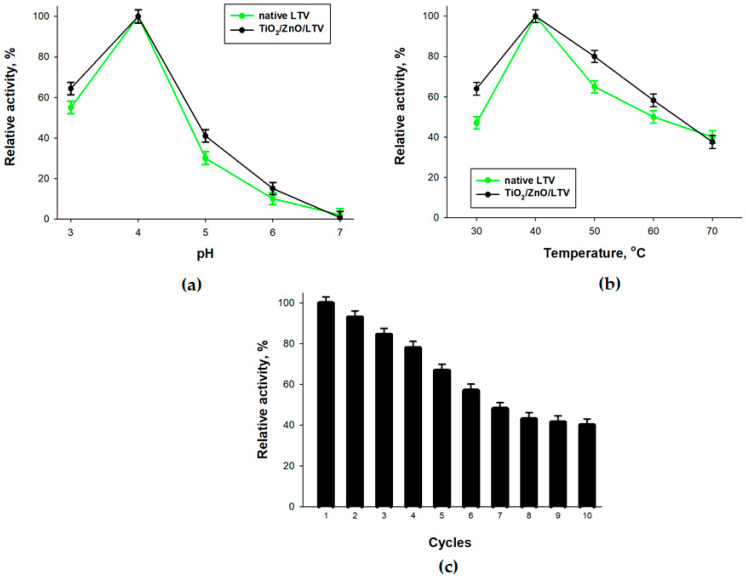
The influence of (**a**) pH, (**b**) temperature, and (**c**) repeated use on the catalytic activity of native enzyme and immobilized laccase.

The influence of pH, temperature and repeated use on the enzymatic activity of the immobilized laccase was determined. The results are presented in Figure 2. From the results shown in Figure 2a, it is concluded that the immobilized laccase exhibited slightly higher activity than its free form in the analyzed range. In both cases, maximal activity was reached at pH 4. Below and above pH 4 the relative activity is lower. A similar situation is observed for variations in temperature (Figure 2b). The maximum enzymatic activity of both free and immobilized forms of LTV occurs at 40 °C. It is notable that the relative activity of the TiO_2_/ZnO/LTV system remains above 60% in the temperature range from 30 to 60 °C.

The heterogeneous form of immobilized LTV is the one of the most significant advantages of the immobilization process. It means that the laccase that is immobilized on TiO_2_/ZnO can be used over several enzymatic reaction cycles. In this study, 10 cycles of the ABTS oxidation reaction were carried out in the presence of the TiO_2_/ZnO/LTV biocatalyst. The results (Figure 2c) show that after 10 cycles the biocatalytic system still retained ca. 40% of its initial activity. Similar results were obtained when different supports based on TiO_2_ and ZnO were used for enzyme immobilization [40,41,42].

### 3.2. Physicochemical Characterization of TiO_2_/ZnO and the TiO_2_/ZnO/LTV Biocatalytic System

**Figure 3:**
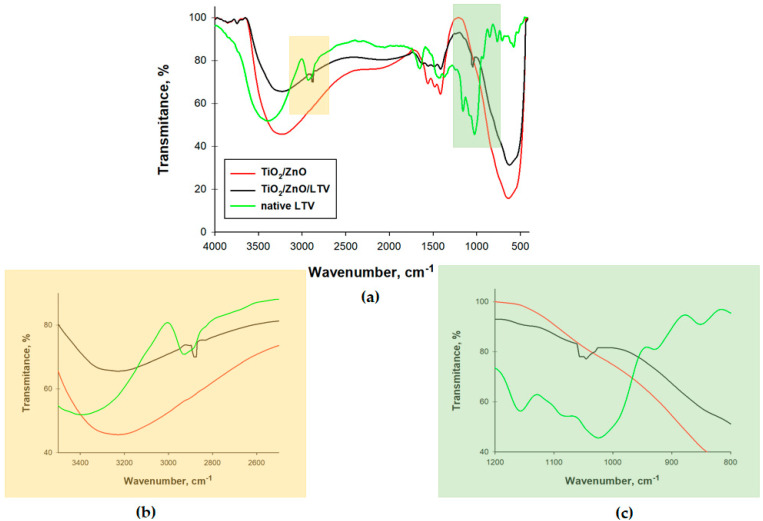
FTIR spectra of TiO_2_/ZnO, native LTV, and immobilized laccase (TiO_2_/ZnO/LTV) (**a**), and two characteristic ranges corresponding to –C–H bonds (**b**) and –C–O bonds (**c**).

Besides typical catalytic evaluation, physicochemical analyses were also used to confirm the efficiency of immobilization of laccase on TiO_2_/ZnO. One of the methods used was FTIR analysis, which identifies characteristic groups on the surface of analyzed samples. These results are presented as FTIR spectra in Figure 3. The spectrum of pure TiO_2_/ZnO contains a wide band in the wavenumber range 3600–3000 cm^−1^, ascribed to the stretching of hydroxyl bonds. The small peak at 1700–1500 cm^−1^ corresponds to water adsorbed by the support. The bands in the range 800–500 cm^−1^ are attributed to the stretching of Zn–O and Ti–O bonds. The peaks on the native LTV spectrum reflect stretching of –NH bonds (3454 cm^−1^), stretching of –C–H bonds (2963 cm^−1^), vibrations of amide I, II and III bonds (1622 cm^−1^, 1489 cm^−1^, 1324 cm^−1^, respectively) and the stretching of –C–O bonds (1146 cm^−1^). The effectiveness of the immobilization is confirmed by the presence of characteristic groups for native LTV and pure TiO_2_/ZnO on the FTIR spectrum of the TiO_2_/ZnO/LTV. On this spectrum, two characteristic ranges are observed. The first (the yellow area) has a peak at 2950 cm^−1^, correlating with the –C–H bonds present in the protein structure. The second (the green area) is a signal derived from the stretching of –C–O bonds (with a maximum at 1046 cm^−1^). These bonds are observed for the enzyme structure, but they are not visible on the spectrum for pure TiO_2_/ZnO.

**Figure 4:**
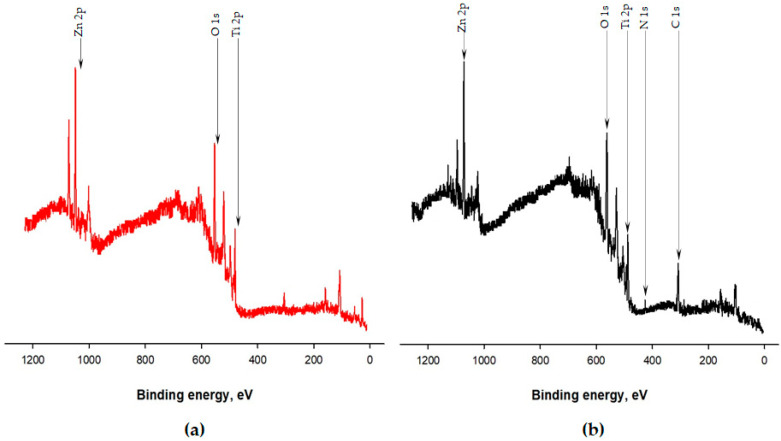
XPS survey spectra of (**a**) TiO_2_/ZnO and (**b**) TiO_2_/ZnO/LTV.

**Figure 5:**
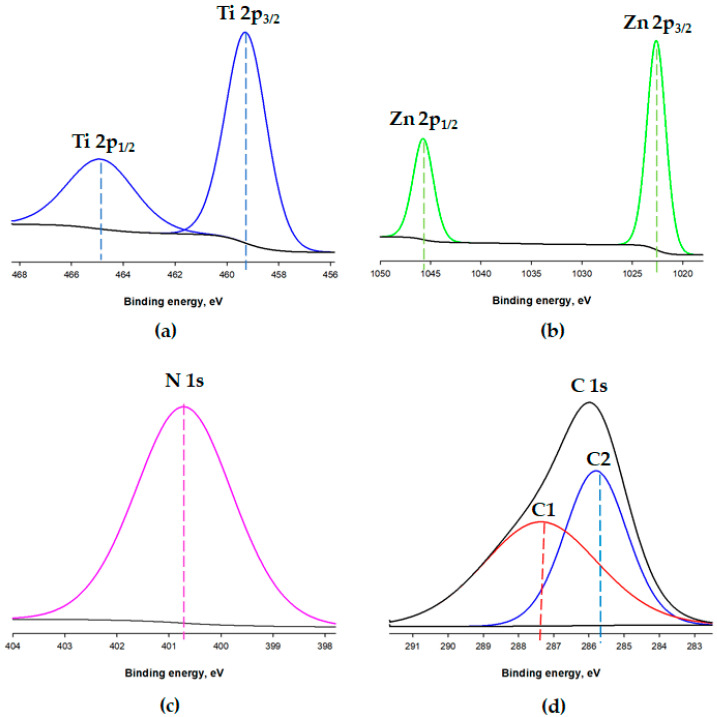
Deconvoluted XPS spectra of (**a**) Ti, (**b**) Zn, (**c**) N and (**d**) C.

X-ray photoelectron spectroscopy (XPS) was exploited to determine the surface composition of the systems before (TiO_2_/ZnO) and after immobilization (TiO_2_/ZnO/LTV). The analyses results of the TiO_2_/ZnO oxide carrier and TiO_2_/ZnO/LTV biocatalytic system chemical composition are presented in Figure 4 and Figure 5. On the surface of the TiO_2_/ZnO oxide system, elements such as titanium, zinc and oxygen are present. The TiO_2_/ZnO/LTV biocatalytic system contains the same elements, but nitrogen and carbon are also present, which indicates the laccase adsorption (Figure 4).

The binding energy values (464.9 and 459.3 eV) determined in the Ti 2p spectrum (Figure 5a) are due to Ti 2p_1/2_ and Ti 2p_3/2_ emissions of Ti^4+^ sites, respectively, while the binding energy values (1045.7 and 1022.6 eV) determined in the Zn 2p spectrum (Figure 5b) are assigned, respectively, to Zn 2p_1/2_ and Zn 2p_3/2_ emissions of Zn^2+^.

Further information about interaction of the enzyme with the TiO_2_/ZnO surface is supplied by an analysis of the N 1s and C 1s lines of the XPS spectrum. The XPS N 1s and C 1s lines of the TiO_2_/ZnO/LTV are shown in Figure 5c,d. Nitrogen atoms, which can be marked with the XPS technique, do not exist on the TiO_2_/ZnO oxide system surface. Therefore, it can be assumed that the N 1s signal for TiO_2_/ZnO/LTV is completely associated with the presence of the enzyme in the sample, and exactly with the incorporation of amino acids in its structure. On the N 1s line (Figure 5c) a symmetrical peak with a binding energy of 400.7 eV is observed. The data available in the literature show that XPS N 1s lines having highest energy, at about 400 eV, is typical for amino acids and proteins, which also confirms that the laccase occurs on the TiO_2_/ZnO surface [47]. Moreover, carbon atoms are also present on the TiO_2_/ZnO/LTV surface, which is probably associated with the laccase structure. Deconvolution of the experimental data was made using a model consisting of C1 and C2 transition. The C1 (287.3 eV) corresponds to a C=O functional group. The component C2, with a binding energy of 285.7 eV, determines the occurring of the C–C/–N groups. All of the above-mentioned groups indicate the presence of laccase on the TiO_2_/ZnO surface [48].

**Figure 6:**
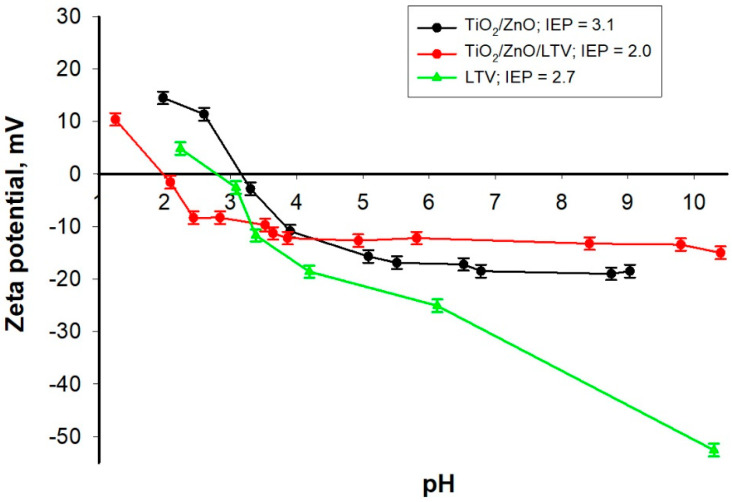
Zeta potential vs. pH of pure TiO_2_/ZnO, TiO_2_/ZnO/LTV and free LTV.

According to Min et al. [49], based on the zeta potential (*ZP*), the electrostatic interactions between an enzyme and its support can be determined, and *ZP* can also be used as an “indicator” of binding efficiency in simple adsorption. Further, the zeta potential was studied as a function of pH, and the isoelectric point (*IEP*) was determined. The results are shown in Figure 6. The free laccase (LTV) has its isoelectric point (*IEP*) at pH 2.7, and its zeta potential ranges from +4.1 to −52.9 mV within the analyzed pH range (2–10). The pure TiO_2_/ZnO exhibits a positive charge in the small pH region 2–3 and a negative charge in the pH region 3.2–10 (the isoelectric point is at pH 3.1). The TiO_2_/ZnO/LTV system shows similar behavior to that of TiO_2_/ZnO without the enzyme, with the isoelectric point shifted to pH 2.0. These results indicate that electrostatic attraction forces between the enzyme and the TiO_2_/ZnO oxide system should be expected [49].

**Figure 7:**
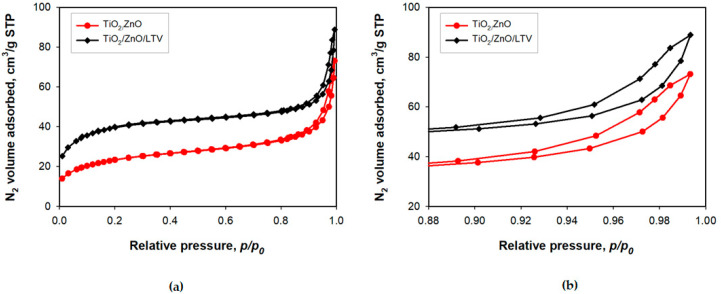
Nitrogen adsorption/desorption isotherms of pure TiO_2_/ZnO and TiO_2_/ZnO/LTV biocatalytic system in different *p/p*_0_ ranges: (**a**) 0.00–1.00, (**b**) 0.88–1.00.

The next part of the presented studies involved the effect of the immobilization process on the value of the surface area (*A_BET_*), pore volume (*V_p_*) and pore size (*S_p_*) (Table 3). The small hysteresis loop of TiO_2_-ZnO covers the relative pressure range *p/p*_0_ = 0.85–1.0. The mean pore diameter of TiO_2_/ZnO is 5.8 nm, and the total pore volume is 0.11 cm^3^/g (Table 3). The quantity of nitrogen adsorbed for relative pressure in the range *p/p*_0_ = 0.18–0.9 mildly increases; above *p/p*_0_ = 0.94, the amount of N_2_ adsorbed quickly grows to achieve the highest value of 73 cm^3^/g at *p/p*_0_ = 1.0. For the TiO_2_/ZnO/LTV biocatalytic system, the volume of nitrogen adsorbed at *p/p*_0_ = 1.0 is higher (90 cm^3^/g), the *S_p_* = 4.3 nm, and the total *V_p_* = 0.14 cm^3^/g. The pure TiO_2_/ZnO has a surface area of 77 m^2^/g, while the surface area of the sample after immobilization (TiO_2_/ZnO/LTV) increases to 126 m^2^/g. The increase in the surface area after immobilization may indicate a low number of pores, because the shape of the isotherms in Figure 7b suggests that mesopores exist only between small particles. Furthermore, the addition of an enzyme on the surface of TiO_2_/ZnO may create heterogeneity and roughness and, therefore, a higher surface area.

### 3.3. Decolorization of Organic Dyes by the TiO_2_/ZnO/LTV Biocatalytic System

**Figure 8:**
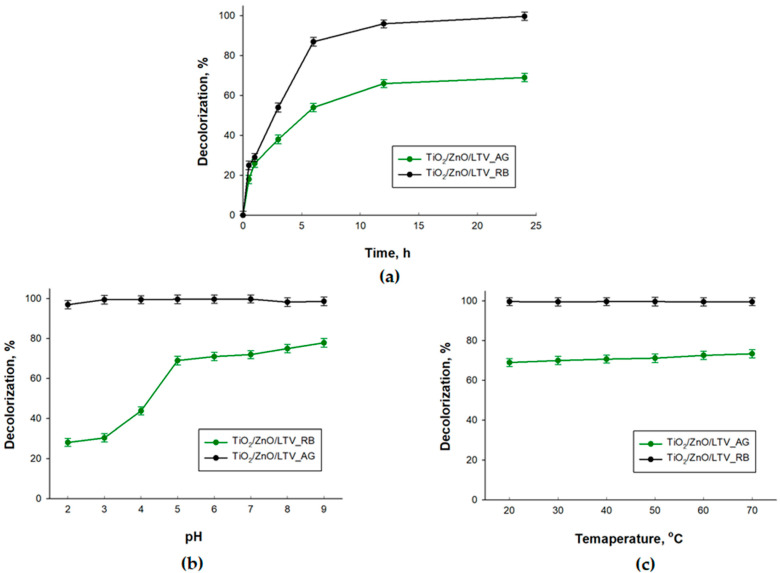
Influence of (**a**) time, (**b**) pH and (**c**) temperature on the decolorization of C.I. Reactive Black 5 and C.I. Acid Green 25.

The proposed TiO_2_/ZnO/LTV biocatalytic system was used to decolorize and degrade the C.I. Reactive Black 5 (RB5) and C.I. Acid Green 25 (AG25) dyes. At this stage, the effect of time, pH and temperature on dye decolorization efficiency (*DE*) was determined. The results are presented in Figure 8. These data show that the decolorization efficiency is higher, over the analyzed parameter ranges, for the RB5 dye than for the AG dye. The decolorization efficiency of RB5 is highest after 24 h, reaching almost 100% (Figure 8a). As can be seen, changes in the pH and process temperature do not have a significant effect on RB5 decolorization (Figure 8b,c). In the whole analyzed range of pH (2–9) and temperature (20–70 °C), the decolorization efficiency is about 99%. Poorer results are obtained for the removal of AG25 dye. In this case, the *DE* is ca. 60% after 24 h. This parameter is also stable with variation in the temperature of the decolorization process. However, variation in the decolorization efficiency is observed at different pH values: the *DE* decreases below pH = 5 and increases above pH = 5. The maximum DE (ca. 80%) is achieved at pH = 9.

The prepared biocatalytic system can be used successfully in the dye decolorization process, in comparison with laccase immobilized on other supports. As shown in Table 4, laccase immobilized on supports based on TiO_2_ or ZnO has been used to remove various dyes (for example: Alizarin Red S, Remazol Brilliant Blue R, Malachite Green, Crystal Violet, Acid Blue etc.) [34,35,36,37]. Those dyes were removed with 60–90% efficiency. Furthermore, the efficiency of the removal of C.I. Reactive Black 5 varied depending on the support used: TiO_2_/ZrO_2_ (15%), Fe_3_O_4_-MWCNT@SiO_2_ (80%), magnetic chitosan nanoparticles (90%), Fe_3_O_4_-NH_2_@MIL-101 (Cr) (65%) [39,50,51,52,53]. It is noteworthy that the biocatalytic system presented in this study also removed C.I. Acid Green 25 (70%). There is no information in the literature about the removal of that type of dye using enzymes.

### 3.4. Biodegradation Products

**Figure 9:**
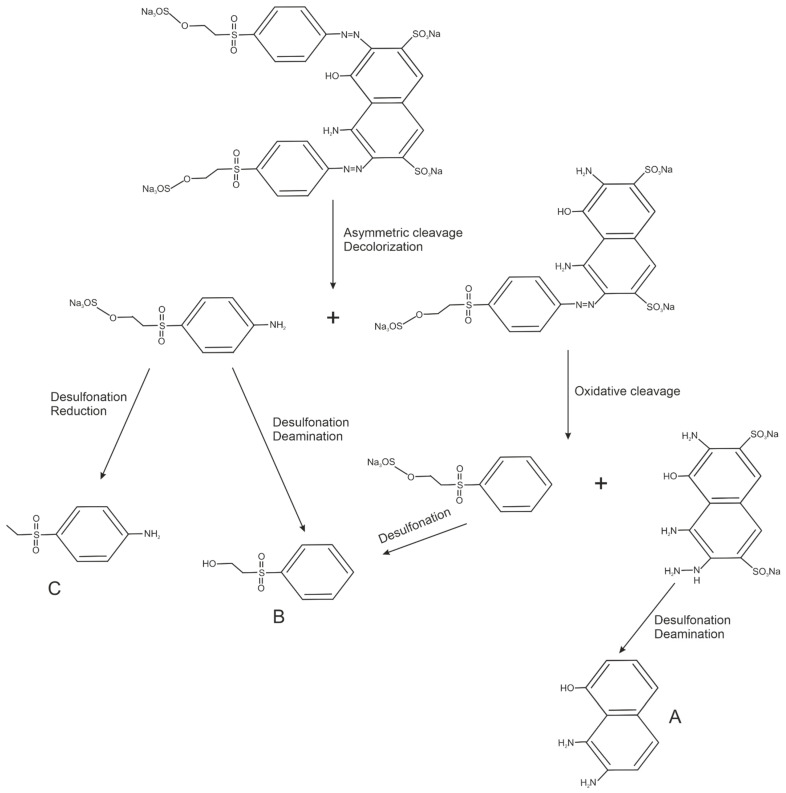
Proposed degradation mechanism of dyes by laccase from *Trametes versicolor* immobilized on TiO_2_/ZnO, on the RB5 example.

Based on the mass spectrometry technique, products of the degradation of RB5 and AG25 by the TiO_2_/ZnO/LTV biocatalytic system were proposed. Possible structures could be defined (Table 5) from the mass spectra and m/z values obtained by MS, as depicted in Figures S1 and S2 (see Supplementary Materials). The MS results suggest that the decolorization of RB 5 proceeded via cleavage of azo bonds (N=N) resulting in the formation of A (m/z = 174), B (m/z = 186) and C (m/z = 185) products, as shown in Table 5. In the case of the AG 25 dye, C–N bond cleavage takes place to produce the compounds D (m/z = 186) and E (m/z = 217) (Table 5). On the basis of the MS results, a supposed dye degradation mechanism was proposed with the example of the RB5 dye (Figure 9). According to the literature, azo dye degradation (RB5) by laccase starts by asymmetric cleavage of the azo bond, followed by oxidative cleavage, desulfonation and deamination [54]. During the degradation of anthraquinone dye, the chromophore of AG25 can be broken down through the C–N cleavage forming smaller molecules. Next, the deamination and oxidation can take place [55]. The suggested degradation compounds and mechanism are in agreement with earlier reports on the degradation of azo and anthraquinone dyes [56,57,58,59].

## 4. Conclusions

The present study has shown that a TiO_2_/ZnO material can be successfully used as a support for laccase. The amount of immobilized laccase (469.6 mg/g_support_) was determined. It was also found that the prepared biocatalytic system (TiO_2_/ZnO/LTV) retains about 40% of its activity in the whole analyzed temperature range and after 10 reaction cycles. The immobilization efficiency was confirmed using several physicochemical analyses (spectroscopic, porous, electrokinetic). The presence of characteristic groups (–C–O and –C–H) in the TiO_2_/ZnO/LTV system indicates the successful incorporation of the enzyme on the TiO_2_/ZnO surface. The efficiency of immobilization is additionally confirmed by XPS analysis (presence of nitrogen and carbon in the XPS spectra of TiO_2_/ZnO/LTV). Moreover, changes in the porous parameter values between pure TiO_2_/ZnO and the TiO_2_/ZnO/LTV biocatalytic system also reflect the effectiveness of immobilization. The differences in the zeta potential and isoelectric point values may suggest the existence of electrostatic interactions between the laccase and the TiO_2_/ZnO carrier. The effective use of the proposed biocatalytic system in the process of removing organic dyes was a key element of the research. The results showed that C.I. Reactive Black 5 and C.I. Acid Green 25 were successfully removed from a water solution (99% and 70%, respectively). Based on MS analysis, the degradation products were established, being the consequence of the cleavage of N=N and C–N bonds in accordance with the general patterns of degradation of similar molecules in water.

## Figures and Tables

**Table 1:** Characterization of dyes.

Name	Class	Chemical Structure
C.I. Reactive Black 5	Azo dye	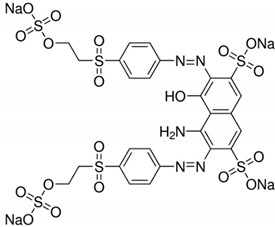
C.I. Acid Green 25	Anthraquinone dye	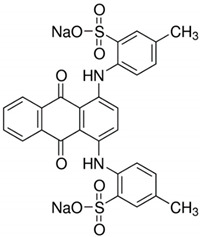

**Table 2:** Results of Bradford analysis.

Immobilization Number	*P*, mg/g_support_	*IY*, %
1	470.4	99.4
2	470.7	99.5
3	468.6	99.4
4	468.9	99.4
Average	469.6 ± 0.9	99.4 + 0.05

**Table 3:** Porous structure parameters of pure TiO_2_/ZnO and TiO_2_/ZnO/LTV biocatalytic systems.

Sample Name	*A_BET_*, m^2^/g	*V_p_*, cm^3^/g	S_p_, nm
TiO_2_/ZnO	77	0.11	5.8
TiO_2_/ZnO/LTV	126	0.14	4.3

**Table 4:** Comparison of the present results with previous studies of the application of immobilized laccase in dye decolorization.

Support	Activity after 10 Cycles	Activity in 3–6 pH Range	Activity in 30–70 °C Temperature Range	Dye Removed	*DE*, %	Ref.
TiO_2_/ZrO_2_	-	-	-	Alizarin Red S	60	[39]
				Remazol Brilliant Blue R	70	
				Reactive Black	15	
TiO_2_/cellulose	30%	>20%	>50%	Reactive Red X-3B	80	[40]
TiO_2_ nanoparticles	-	>20%	>30%	Indigo Carmine	90	[41]
				Alizarin Red	60	
				Trypan Blue	90	
				Malachite Green	90	
TiO_2_/PAN/O-MMT	50%	>20%	>50%	Crystal Violet	80	[42]
ZnO/SiO_2_	-	-	-	Remazol Brilliant Blue R	85	[43]
				Acid Blue 25	80	
ZnO chelated with Cu^2+^	-	-	-	Alizarin Red S	95	[44]
Fe_3_O_4_-MWCNT@SiO_2_	80%	>40%	>70%	Reactive Black	65	[50]
				Acid Red 88	98	
				Eriochrome Black T	99	
Magnetic chitosan nanoparticles	40%	>30%	>50%	Acid Red 37	90	[51]
				Reactive Black 5	90	
				Evans Blue	90	
				Direct Blue T	90	
Fe_3_O_4_-NH_2_@MIL-101(Cr)	-	>70%	>50%	Alizarin Red S	90	[52]
				Reactive Black 5	80	
TiO_2_/ZnO	45%	>20%	>40%	Reactive Black 5	99	This study
				Acid Green 25	70	

**Table 5:** Possible structures of degradation products of C.I. Reactive Black 5 and C.I. Acid Green 25, based on MS analysis.

Dye	Compound	Chemical Structure	Chemical Formula	m/z *, Da
C.I. Reactive Black 5	A	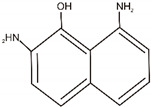	C_10_H_10_ON_2_	175
	B	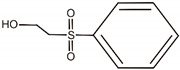	C_8_H_10_O_3_S	187
	C	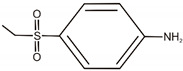	C_8_H_11_O_2_NS	185
C.I. Acid Green 25	D	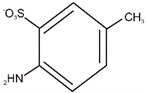	C_8_H_7_O_3_NS	186
	E	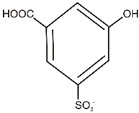	C_7_H_5_O_6_S	217

* m/z—a mass of ions presence on the MS spectra: (M + 1) for degradation products of RB5 (A, B and C) and (M − 1) for degradation products of AG25 (D and E).

## Data Availability

Not applicable.

## Supplementary Materials

The following are available online at https://www.mdpi.com/article/10.3390/ma14206030/s1, Figure S1: MS spectra of (a) initial dye solution and (b) solution after degradation of C.I. Reactive Black 5, Figure S1: MS spectra of (a) initial dye solution and (b) solution after degradation of C.I. Acid Green 25.
