# Supplementary material for: Catalytic and Physicochemical Evaluation of a TiO2/ZnO/Laccase Biocatalytic System: Application in the Decolorization of Azo and Anthraquinone Dyes
Source: Materials (Basel). 2021 Oct 13;14(20):6030. doi: 10.3390/ma14206030 (PMC8537205; doi:10.3390/ma14206030)
Supplement: Supplementary file 1 [file materials-14-06030-s001.zip › materials-1399351-supplementary.pdf]

Suplementary materials (SM)

Catalytic and Physicochemical Evaluation of a TiO_2/ZnO/Laccase Biocatalytic System: Application in the Decolorization of *Azo* and *Anthraquinone* Dyes

Agnieszka Kołodziejczak-Radzimska [1,*], Joanna Zembrzuska [2], Katarzyna Siwińska-Ciesielczyk [1], and Teofil Jesionowski [1]

[1] Institute of Technology and Chemical Engineering, Faculty of Chemical Technology, Poznan University of Technology, Berdychowo, PL-60965 Poznan, Poland; katarzyna.siwinska-ciesielczyk@put.poznan.pl (K.S.-C.); teofil.jesionowski@put.poznan.pl (T.J.)

[2] Institute of Chemistry and Technical Electrochemistry, Faculty of Chemical Technology, Poznan University of Technology, Berdychowo, PL-60965 Poznan, Poland; joanna.zembrzuska@put.poznan.pl

* Correspondence: Agnieszka.kolodziejczak-radzimska@put.poznan.pl

MS spectra

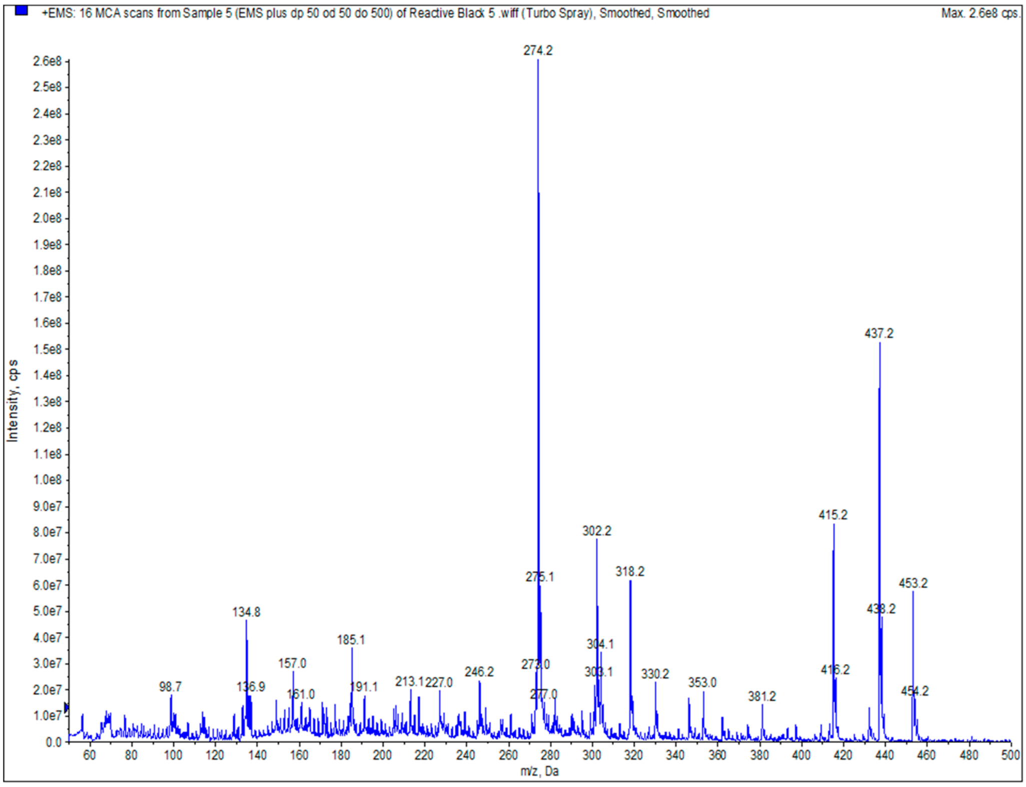

(a)

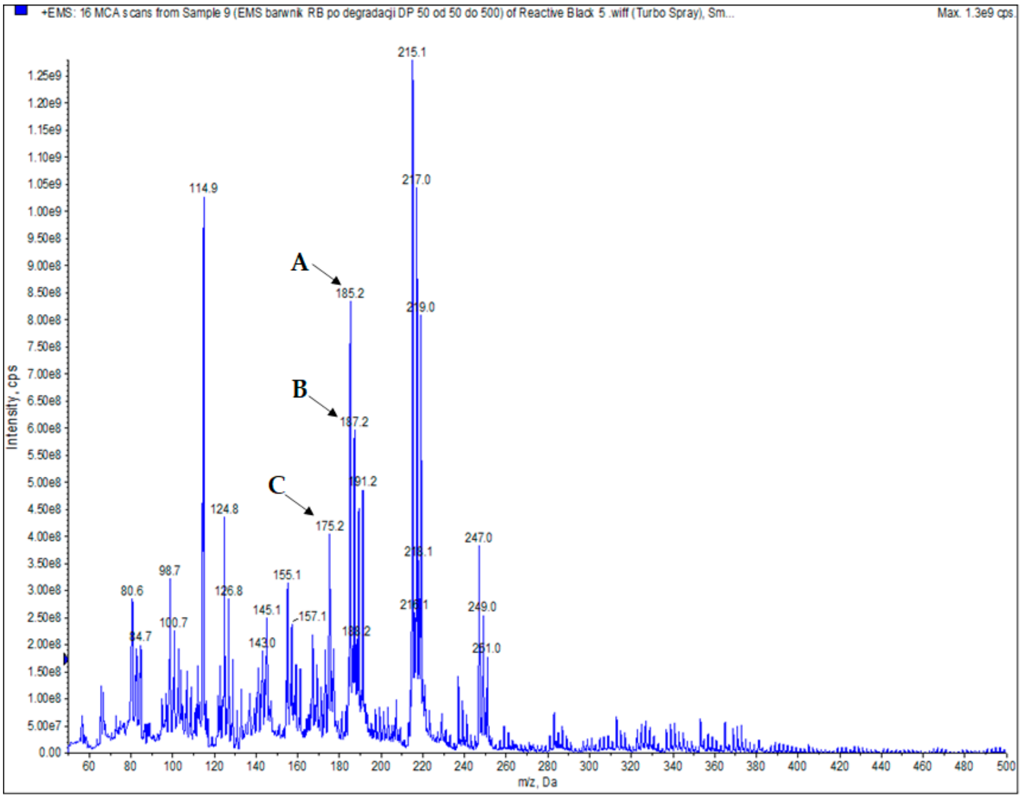

(**b**)

Figure S1. MS spectra of (**a**) initial dye solution and (**b**) solution after degradation of *C.I. Reactive Black 5***.**

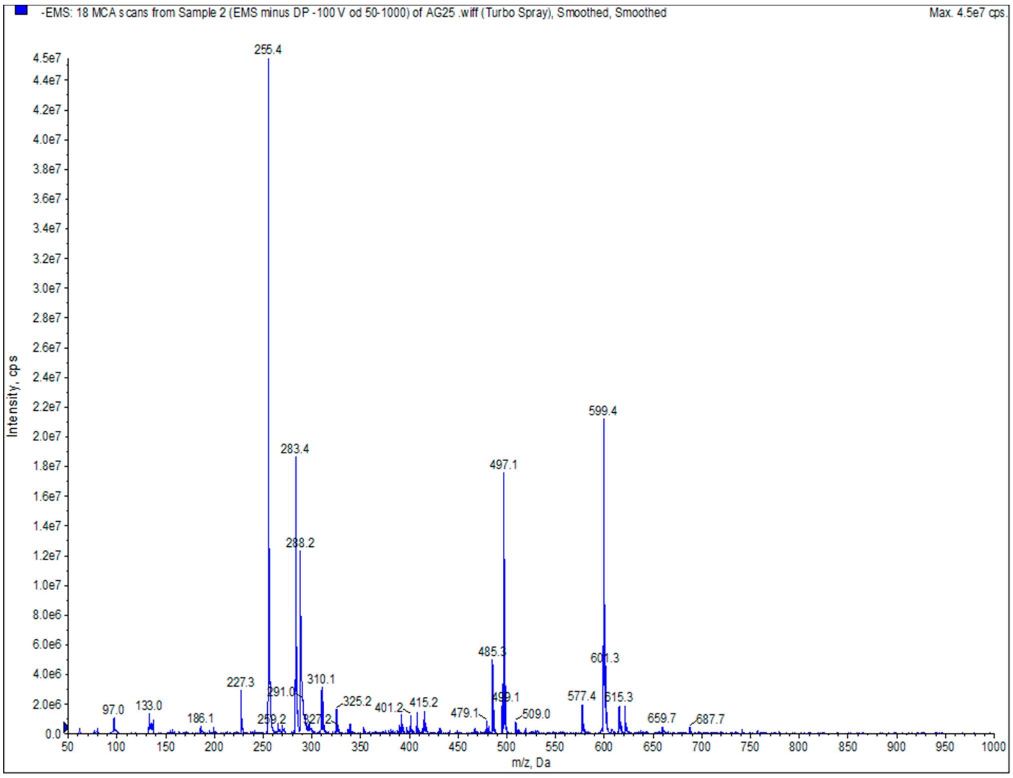

(a)

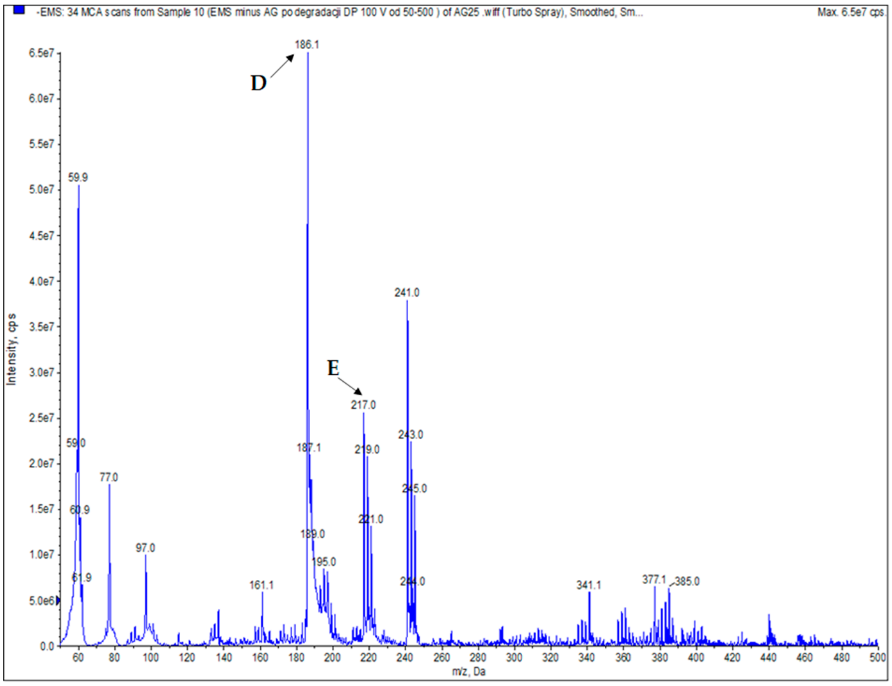

(b)

Figure S2. MS spectra of (**a**) initial dye solution and (**b**) solution after degradation of *C.I. Acid Green 25* **.**
